# Electrodeposition of High-Quality Ni/SiC Composite Coatings by Using Binary Non-Ionic Surfactants

**DOI:** 10.3390/molecules28083344

**Published:** 2023-04-10

**Authors:** Han Rao, Weiping Li, Fuzhen Zhao, Yongfa Song, Huicong Liu, Liqun Zhu, Haining Chen

**Affiliations:** National Experimental Teaching Demonstration Center for Materials Science and Engineering, School of Materials Science and Engineering, Beihang University, No. 37 Xueyuan Road, Beijing 100191, China

**Keywords:** Ni/SiC composite coatings, co-electrodeposition, binary non-ionic surfactants, nanoparticle agglomeration, wear resistance

## Abstract

In order to increase the hardness, wear resistance and corrosion resistance of nickel-based coatings, pure nickel is often co-electrodeposited with silicon carbide (SiC) particles. However, SiC particles tend to agglomerate and precipitate in the bath, which reduces the amounts of nanoparticles and causes nonuniformity. Herein, we solve these problems by using binary non-ionic surfactants (Span 80 and Tween 60) to effectively disperse SiC particles (binary-SiC) in the bath, which suppresses nanoparticles agglomeration and leads to uniformly distributed SiC particles in the composite coatings. In comparison to the Ni/SiC coatings electrodeposited from the commonly used SDS-modified SiC, the coatings prepared with binary-SiC (Ni/binary-SiC) show finer crystallization and a smoother surface. In addition, the Ni/binary-SiC coatings exhibit higher hardness (556 Hv) and wear resistance (2.95 mg cm^−2^). Furthermore, higher corrosion resistance is also achieved by the Ni/binary-SiC coatings.

## 1. Introduction

Electroplated nickel is an extensively implemented industrial protective coating that protects various components from corrosion and wear. However, due to its low strength and hardness, pure nickel cannot effectively protect parts. Silicon carbide (SiC) is a potential functional material with high hardness, high thermal conductivity and high stability, and has been widely used in optical devices, nanotechnology and nuclear material science [1,2,3]. To enhance strength, pure nickel is usually co-deposited with SiC nanoparticles to fabricate composite coatings [4,5,6]. In the process of composite electroplating with nanoparticles, the most concerning problem is that nanoparticles tend to agglomerate and precipitate, which decreases the nanoparticle content and leads to nonuniformity in composite coatings [7].

To solve the above problems, surfactants are usually added to the bath to change the surface hydrophobicity of particles [8]. For example, Yan et al. [9] used sodium dodecyl sulfate (SDS) to disperse SiC nanoparticles in a Ni bath, and a uniform Ni/SiC composite coating was obtained that achieved a hardness of 407.76 Hv. Ger et al. [10] reported that the addition of cetyltrimethylammonium bromide (CTAB) could reduce the agglomeration of SiC particles in the plating bath and improve the hardness of Ni/SiC composite coatings. Kan et al. [11] have compared the effects of SDS and CTAB on the properties of Ni/SiC coatings. It was found that CTAB could increase the content of SiC nanoparticles but induce poor uniformity due to agglomeration. Although ionic surfactants could increase the content of nanoparticles, the quality of the composite coatings still needed to be improved before practical application [12,13].

By far, many typical surfactants have been used to disperse nanoparticles, including SDS [14], hexadecylpyridinium bromide (HPB) [15,16], CTAB [17] and so on. However, dispersed nanoparticles made by using ionic surfactants usually tend to re-agglomerate after being added to the electroplating solution [12]. Compared with ionic surfactants, non-ionic surfactants and their combined surfactants are less affected by the ions in the electrolyte solution [18]. Therefore, non-ionic surfactants seem to be more promising than ionic surfactants for dispersing nanoparticles. Nevertheless, previous studies mainly used a single non-ionic surfactant, whose effect is not enough for practical applications [19,20,21,22].

Herein, we made an attempt to use binary non-ionic surfactants (Span 80 and Tween 60) to disperse SiC particles in a Ni bath, and the Ni/SiC composite coatings with uniformly distributed SiC particles were electrodeposited. In comparison to the Ni/SiC coatings electrodeposited from the commonly used SDS-modified SiC, the coatings prepared with binary-SiC (Ni/binary-SiC) show finer crystallization and a smoother surface. In addition, the Ni/binary-SiC coatings exhibit higher hardness (556 Hv) and wear resistance (2.95 mg cm^−2^). Furthermore, higher corrosion resistance is also achieved by the Ni/binary-SiC coatings.

## 2. Results and Discussion

### 2.1. Modification and Dispersion of SiC Particles

**Figure 1:**
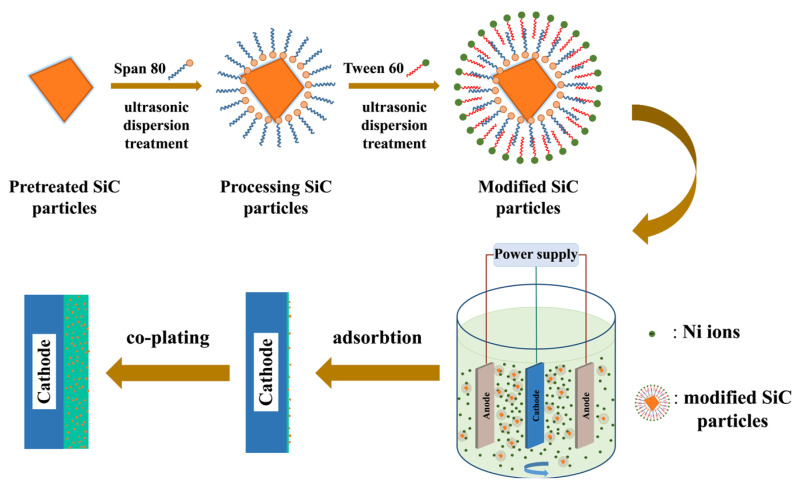
Schematic illustrating the modification of SiC particles and the co-plating of Ni/SiC coatings.

Before being added to the bath, SiC nanoparticles were treated with Span 80 and Tween 60, as illustrated in Figure 1. The Hydrophile-Lipophilic Balance (HLB) value of the surfactant represents the ratio of the hydrophilic group to the oleophilic group, which reflects its hydrophilicity. The higher the HLB value, the better the hydrophilicity of surfactant molecules [23]. The fine dispersion effect of binary surfactants is attributed to the different HLB values of Span 80 and Tween 60. The HLB values of Span 80 and Tween 60 are 4.3 and 14.5, respectively. Although the non-polar carbon chain lengths of the two surfactants are similar, Tween 60 molecules have more hydrophilic groups than Span 80 molecules. Hence, hydrophobic long chains of Tween 60 molecules could be easily combined with the hydrophobic long chains of Span 80 molecules and hydrophilic groups combined with external water molecules. Such a bilayer structure is conducive to the reduction of free energy in the system [24,25,26,27,28,29]. Consequently, it is reasonable to speculate on the formation of a bilayer coating structure at sequential modification of Span 80 and Tween 60, which facilitates more uniform dispersion of SiC particles. Well-dispersed nanoparticles are more conducive to co-deposition. According to the Guglielmi two-step adsorption theory [30], the modified SiC particles first move to the cathode surface for weak adsorption and are then co-deposited with the reduced metal to form a composite coating, as depicted in Figure 1. Agglomerated particles will be subject to a greater force field in the weak adsorption stage and are more likely to be washed into the solution, which makes it difficult to be co-deposited in the coating [10].

**Figure 2:**
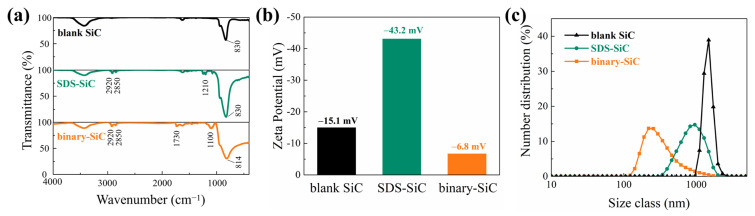
(**a**) Fourier-transform infrared spectra, (**b**) zeta potential, and (**c**) particle size distribution of blank SiC, SDS-SiC and binary-SiC.

In order to evaluate the absorption of surfactants on SiC particles, Fourier-transform infrared (FTIR) spectra were collected. As shown in Figure 2a, the peak at 830 cm^−1^ for blank SiC corresponds to the Si-C bond [31]. Two absorption peaks at 2920 cm^−1^ and 2850 cm^−1^ for the SiC particles modified with SDS (SDS-SiC) and binary surfactants (binary-SiC) could be detected, which could be indexed to the -CH_2_- and -CH_3_ groups [32]. In SDS-SiC, the peak at 1210 cm^−1^ corresponds to the sulfate group, which illustrates that SiC particles are successfully modified by SDS. In binary-SiC, the absorption peak at 1100 cm^−1^ is related to the unique functional groups of C-O-C of Tween 60. Meanwhile, the significant transmittance differences in the range of 400–800 cm^−1^ in binary-SiC may be attributed to the absorption of cis C=C groups (665–770 cm^−1^) for Span 80, which illustrates the adsorption of Span 80 on the SiC particle surface [33].

As presented in Figure 2b, the zeta potentials of blank SiC, SDS-SiC, and binary-SiC are −15.1 mV, −43.2 mV and −6.8 mV, respectively. The ionization of acidic ≡Si-OH groups on the surface of SiC nanoparticles will generate ≡Si-O^−^ and H^+^ ions in an aqueous solution, which results in a negative zeta potential for blank SiC [34]. For SDS-SiC, the adsorption of dodecyl sulfate ions makes the zeta potential more negative. However, the zeta potential of SiC nanoparticles becomes more positive after the modification of Span 80 and Tween 60, which is attributed to the shielding effect of the adsorbed neutral surfactant molecules [35,36]. Generally, for ionic surfactants modified particles, the higher the absolute value of zeta potential, the greater the electrostatic repulsion between particles, and the better the physical stability. However, the situation will be different in the presence of non-ionic surfactants [24]. Non-ionic surfactants such as Span 80 and Tween 60 mainly rely on the steric hindrance effect to achieve dispersion. Even if the zeta potential of the dispersion system is small, it can maintain good stability and reduce agglomeration. Figure 2c shows the particle size distribution of blank SiC, SDS-SiC and binary-SiC. The average sizes of blank SiC, SDS-SiC and binary-SiC are 1601 nm, 959 nm and 544 nm, respectively. Some semi-blank experiments had also been conducted, as shown in Fig.S5, which confirms the highest dispersibility of binary-SiC. Therefore, consecutive treatments in the correct order by binary surfactants contributed to the dispersion of SiC particles. Based on the dispersion theory of nanoparticles, it could be inferred that long chains of non-ionic surfactants at the adsorption bilayer outside make SiC particles that effectively prevent agglomeration through the steric hindrance [37,38]. The combined treatments with the binary surfactants may be conducive to the formation of a stable adsorption bilayer and the interaction of steric hindrance theoretically; thus, binary-SiC presented an apparent size reduction and more uniform size distribution [26,39].

### 2.2. Phase Structure and Grain Size

**Figure 3:**
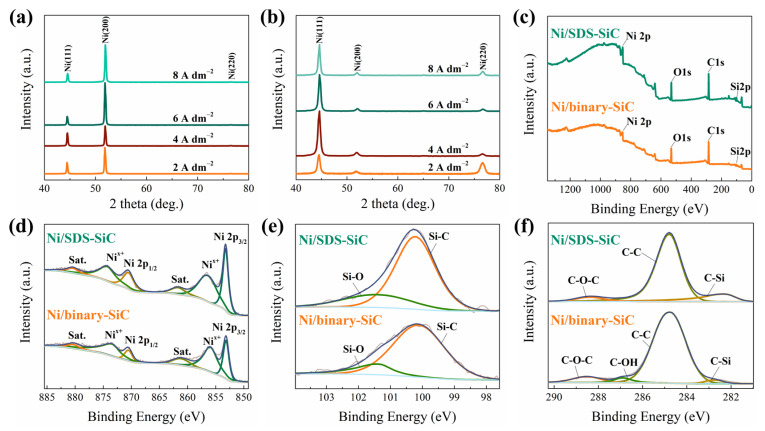
Structure characterization. XRD patterns of composite coatings, (**a**) Ni/SDS-SiC, (**b**) Ni/binary-SiC. XPS spectra of composite coatings at the current density of 2.0 A dm^−2^, (**c**) survey spectra, (**d**) Ni 2p XPS spectra, (**e**) Si 2p XPS spectra, (**f**) C 1s XPS spectra.

XRD and XPS measurements were further conducted to evaluate the phase structure and composition of different coatings. As shown in Figure 3a,b, the XRD patterns are similar for the Ni/SDS-SiC and Ni/binary-SiC coatings. The diffraction peaks at 44.5°, 51.8° and 76.4° could be indexed to the (111), (200) and (220) planes of face-centered cubic-structured Ni (JCPDS no. 87-0712), respectively. The texture coefficients of different crystal planes were calculated according to the texture coefficient formula (Equation (1)) and the results are listed in Table 1.

(1) $${TC}_{\left(hkl\right)}=\frac{I_{\left(hkl\right)}/I_{0(hkl)}}{\sum_{n}I_{n(hkl)}/I_{0(hkl)}}\times 100\%$$

where *I*_(*hkl*)_ is the peak intensity obtained from the sample and *I*_0(*hkl*)_ is the corresponding peak intensity of the standard Ni pdf card (JCPDS no. 87-0712). As indicated, the preferentially oriented crystal plane is (200) for the Ni/SDS-SiC coating, while it changes to (111) for the Ni/binary-SiC coating. As proved in the literature, the composite coatings with the preferentially oriented crystal plane of (200) usually show lower hardness and higher ductility than that of (111) [40]. The grain size was calculated by Scherrer’s formula (Equation (2)):

*D* = *Kλ/Bcosθ* (2)

where *D* is the average thickness of the grain perpendicular to the crystal plane, *B* is the width of the half peak height of the diffraction peak of the measured sample, *θ* is the Bragg angle, *λ* is the X-ray wavelength (1.5406 Å) and *K* is the Scherrer constant. As shown in Table 1, the grain size of the Ni/binary-SiC coating gradually increases with current density, which could be attributed to grain growth induced by concentration polarization at high current density [40]. Compared with the Ni/SDS-SiC coating, the grain size is obviously reduced for the Ni/binary-SiC coating, highlighting the great influence of SiC nanoparticles and surfactants on the crystallization of Ni [41].

XPS spectra in Figure 3c confirm the existence of Ni, Si and C in the composite coatings. In Figure 3d, the XPS spectra show a strong peak at 853.2 eV, corresponding to the 2p_3/2_ region of metallic Ni, while the two peaks at 856.7 eV and 861.9 eV correspond to oxidized Ni and a shakeup satellite (Sat.) peaks, respectively. The existence of the oxidized Ni might be attributed to the partial oxidization of Ni at the surface [42]. The Si spectra in Figure 3e show peaks at 100.3 eV and 101.6 eV, which could be indexed to the Si 2p peaks of Si-C and Si-OH species, respectively. The Si-C species most likely comes from SiC particles, while the Si-OH species might come from the ≡Si-OH groups at the surface of SiC particles. The peaks in the C XPS spectrum of the Ni/binary-SiC coating (Figure 3f) at 282.4 eV, 284.8 eV, 286.9 eV and 288.4 eV could be attributed to C-Si, C-C, C-OH and C-O-C species, respectively, which should be due to the absorption of binary surfactants on SiC particles. In contrast, no peak corresponding to C-OH is observed for the Ni/SDS-SiC coating because of the absence of the C-OH functional group in SDS.

**Figure 4:**
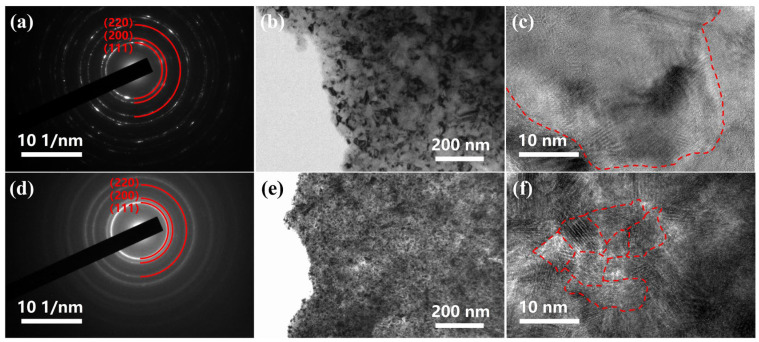
TEM characterization. (**a**) SAED pattern, (**b**) TEM image, (**c**) HRTEM image of Ni/SDS-SiC coating prepared at the current density of 2.0 A dm^−2^; (**d**) SAED pattern, (**e**) TEM image, (**f**) HRTEM image of Ni/binary-SiC coating prepared at the current density of 2.0 A dm^−2^.

TEM images were taken to observe the microstructure and crystal morphology of the Ni/SiC composite coatings. As illustrated in Figure 4a,d, the SAED patterns of the composite coatings show a ring shape, indicating the polycrystalline feature [43]. Compared to the Ni/SDS-SiC coating, the Ni/binary-SiC coating shows more concentric diffraction rings with smaller and darker spots, indicating a finer grain. Figure 4b,e also show the polycrystalline morphology of the composite coatings. According to the high-resolution TEM (HRTEM) image in Figure 4c,f, the average grain size of the Ni/SDS-SiC coating is calculated to be about 36 nm, while it is reduced to about 15 nm for the Ni/binary-SiC coating (Figure S6). Therefore, the TEM results are well consistent with the XRD results.

### 2.3. Morphology of Composite Coatings

**Figure 5:**
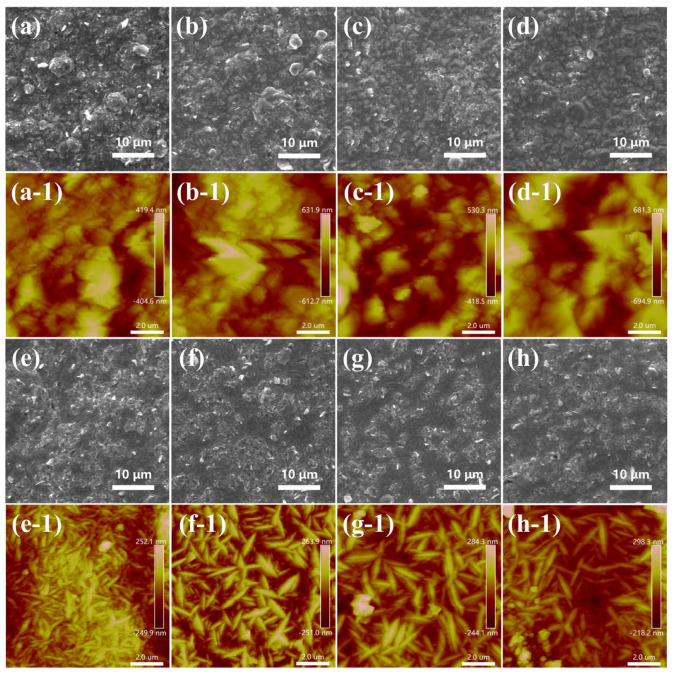
SEM characterization. (**a**–**d**) surface morphology of Ni/SDS-SiC coatings at current densities of 2.0, 4.0, 6.0 and 8.0 A dm^−2^; (**e**–**h**) surface morphology of Ni/binary-SiC coatings at current densities of 2.0, 4.0, 6.0 and 8.0 A dm^−2^. AFM characterization. (**a-1**–**d-1**) AFM images of Ni/SDS-SiC coatings at current densities of 2.0, 4.0, 6.0 and 8.0 A dm^−2^; (**e-1**–**h-1**) AFM images of Ni/binary-SiC coatings at current densities of 2.0, 4.0, 6.0 and 8.0 A dm^−2^.

SEM images are also taken to evaluate the surface morphology of composite coatings. As shown in Figure 5a–d, the surface of the Ni/SDS-SiC coatings is covered with large and disorderly cauliflower-shaped crystals. The maximal content of SiC particles in Ni/SDS-SiC coatings is 3.9 wt% at the current density of 4.0 A dm^−2^ (Table S1). However, the surface morphology of Ni/binary-SiC coatings in Figure 5e–h becomes smoother, and the highest SiC content is 10.8 wt% obtained at the current density of 2.0 A dm^−2^ (Table S1). With the current density increasing, the deposition of Ni was accelerated, while the deposition rate of SiC particles decreased, leading to a reduction in the SiC content in the Ni/binary-SiC coatings. The difference in the morphology and SiC content between the two composite coatings might be attributed to the dispersion effect of different surfactants. SiC particles modified by binary surfactants are less likely to agglomerate, which results in facilitating more flat and even coatings [44]. To further observe the surface morphology, AFM images (Figure 5a-1–h-1) were taken. The Ni/binary-SiC coatings present lower surface roughness, and the maximum value is about 62 nm, much lower than that of the Ni/SDS-SiC coatings (about 168 nm) (Table S2).

### 2.4. Hardness and Wear-Resisting Properties

**Figure 6:**
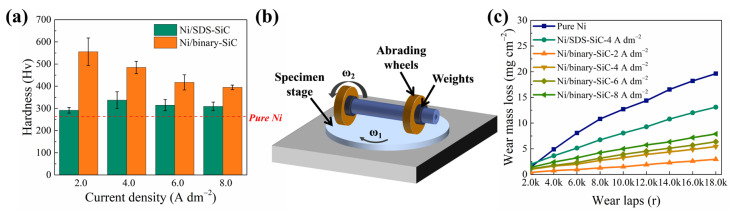
(**a**) Microhardness of the Ni/SDS-SiC and Ni/binary-SiC coatings, (**b**) schematic illustrating the abrasive resistance test, and (**c**) wear loss weight curves of the Ni/SDS-SiC and Ni/binary-SiC coatings.

The micro-hardness and wear resistance of different composite coatings are evaluated. As shown in Figure 6a, the micro-hardness of the pure nickel coating is 263 Hv. The Ni/SDS-SiC coatings show slightly higher micro-hardness (337 Hv) than the pure nickel coating. Interestingly, the hardness of Ni/binary-SiC coatings is greatly improved, and the maximal hardness reaches 556 Hv at the current density of 2 A dm^−2^. The hardness of composite coatings gradually reduces with the current density, which may be attributed to fewer SiC particles being co-deposited on the surface [6,45].

To evaluate the wear resistance of composite coatings, weight loss data was collected before and after the wear test. As indicated in Figure 6c, with the increase in wear cycles, the wear loss of coatings gradually increases, and the relationship is approximately linear. Wear loss is suppressed for the composite coatings compared with the pure nickel coating, and the Ni/binary-SiC coating shows the highest effect. For the Ni/binary-SiC coating, the higher current density would lead to larger wear loss, and the best wear resistance was obtained at 2 A dm^−2^.

The excellent hardness and wear resistance of Ni/binary-SiC coatings may be attributed to the following reasons: on one hand, according to the Hall—Petch formula, grain refinement would lead to an increase in the density of grain boundaries, which would suppress dislocation movement in the plastic deformation of materials and hence improve the hardness and wear resistance. On the other hand, the Ni/binary-SiC coatings contain a larger amount of SiC particles, which will become the main site for carrying the load once subjected to external mechanics or friction [46].

### 2.5. Corrosion Resistance

**Figure 7:**
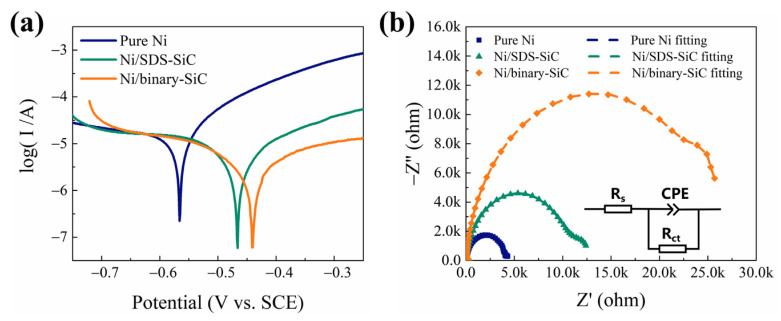
Electrochemical characterizations of composite coatings. (**a**) Tafel plots and (**b**) Nyquist plots. Inset is the electrical equivalent circuit.

Tafel plots (Figure 7a) were taken, and some important parameters were calculated (Table 2). As indicated, the corrosion potential and current of the composite coatings are more positive and smaller, respectively, than those of the pure nickel coating. Furthermore, these two parameters for the Ni/binary-SiC coating are superior to those for the Ni/SDS-SiC coating. The EIS spectra in Figure 7b also confirm the higher corrosion resistance for the composite coatings than that for the pure nickel coating, and the Ni/binary-SiC coating demonstrates the best performance. By fitting the EIS spectra using the electrical equivalent circuit (EEC, inset in Figure 7b), some important parameters were obtained and shown in Table 3. The Rct of the Ni/binary-SiC coating is 26.40 kΩ cm^2^, which is far higher than that of pure nickel and Ni/SDS-SiC and reveals better corrosion resistance. The evolutions in corrosion resistance of composite coatings are closely related to SiC content [47]. From a thermodynamic point of view, its chemical properties are more stable than the metal/alloy matrix. Therefore, the presence of SiC nanoparticles may reduce the effective conductive surface area in the corrosion process, thus improving the corrosion resistance [48,49].

## 3. Materials and Methods

### 3.1. Materials

Nickel sulfate (NiSO_4_·6H_2_O), sorbitan oleate (Span 80, C_24_H_44_O_6_), polyoxyethylene sorbitan monostearate (Tween 60, C_64_H_126_O_26_), sodium dodecyl sulfate (SDS, C_12_H_25_NaO_4_S) and sodium hydroxide (NaOH) were purchased from Shanghai Macklin Biochemical Co., Ltd. SiC powder (irregular shape, 500 nm, α-SiC), nickel chloride (NiCl_2_·6H_2_O), boric acid (H_3_BO_3_), sodium carbonate (Na_2_CO_3_) and trisodium phosphate (Na_3_PO_4_·12H_2_O) were purchased from Shanghai Aladdin Bio-Chem Technology Co., Ltd. Sulfuric acid (H_2_SO_4_) was purchased from Modern Oriental (Beijing) Technology Development Co., Ltd. High purity nickel plate (99.99%) was purchased from Qinghe Shenghang Metal Materials Co., Ltd.

### 3.2. Electrodeposition

The SiC powder was pre-treated before being added to the bath. First, SiC powder was degreased with absolute ethanol. Then acid washing was carried out with 15% diluted sulfuric acid for activation for 1 h. After filtration and separation, the SiC powder was washed with deionized water to neutralize. Then, 0.2 g L^−1^ surfactants (for binary surfactants, 0.2 g L^−1^ Span 80, and the ratio of Span 80 and Tween 60 is 1:1 in molar ratio) were added, and ultrasonic treatment was applied for 10 min. Finally, the dispersed SiC suspensions were slowly added to the Watts plating solution, stirred for 2 h and dispersed by ultrasonic treatment for 10 min. The Watts plating solution contains 300 g L^−1^ NiSO_4_·6H_2_O, 45 g L^−1^ NiCl_2_·6H_2_O and 35 g L^−1^ H_3_BO_3_.

The co-electrodeposition of the Ni/SiC composite coatings was conducted as follows: (1) A carbon steel plate with a size of 20 × 50 mm^2^ was degreased in an alkaline chemical solution containing 90 g L^−1^ NaOH, 40 g L^−1^ Na_2_CO_3_ and 40 g L^−1^ Na_3_PO_4_·12H_2_O, then washed with 15% diluted sulfuric acid to remove oxides before plating. (2) A carbon steel plate and a 99.99% high-purity nickel plate with a larger size of 60 × 70 mm^2^ were used as the cathode and anode, respectively. (3) The pH of the bath was adjusted with an appropriate amount of diluted sulfuric acid to about 4 and the stirring rate was controlled at 400 r/min. The co-electrodeposition was implemented by using a direct current power device at a current density of 2.0, 4.0, 6.0 and 8.0 A dm^−2^. During co-electrodeposition, the temperature and duration were set at 40 °C and 15–60 min, respectively.

### 3.3. Characterizations

Size distribution data and zeta potential distribution data were collected on a Zetasizer particle size analyzer (ZEN3700, Malvern Panalytical Ltd., Malvern, UK). Due to the influence of the refractive index of the plating bath, measurements are carried out in a 1:1 diluted composite plating solution to obtain accurate results. Fourier-transform infrared (FT-IR) spectra were measured on a Nicolet IS10 infrared spectrometer to evaluate the modification effect of binary surfactants.

X-ray diffraction (XRD) patterns were obtained on a D/max-2500 diffractometer at 40 kV and 200 mA with Cu Kα (λ = 1.5406 Å) radiation. The morphology was observed via scanning electron microscopy (SEM, SUPRA 55, Carl Zeiss, Oberkochen, Germany). XPS (Thermo Scientific Escalab 250Xi, California, USA) was used to determine chemical compositions. An atomic force microscope (AFM, Bruker Dimension Icon system, Rheinstetten, Germany) was employed to characterize the variation in the fluctuation of the composite coatings. A transmission electron microscope (TEM, JEM-2100F, NIDEC CORPORATION, Tokyo, Japan) was used to investigate the microstructure of the composite coatings through high-resolution TEM images and selected area electron diffraction patterns.

The hardness of the composite coatings was measured on the surface by a Micro Vickers Hardness Tester (FM810, FUTURE-TECH, Kawasaki, Japan) at 100 g for 15 s. The abrasive resistance of the composite coatings was characterized by an dry abrasion machine (LC-802B, Lichuan, Dongguan, China) with a 500 g normal load and a wear area of about 5.73 cm^2^. The mass loss was calculated by measuring the mass of the composite coatings before and after friction. Tafel plots and EIS spectra (vs. SCE) were obtained in a 3.5 wt% NaCl solution on an electrochemical workstation (CHI 706E, Chenhua, Shanghai, China) by using a three-electrode cell.

## 4. Conclusions

We have used Span 80 and Tween 60 to disperse SiC particles in the Ni bath, and a composite coating (a Ni/binary-SiC coating) was successfully prepared by electrodeposition. Compared with the Ni/SDS-SiC coating, more SiC particles (10.8 wt%) were incorporated in the Ni/binary-SiC coating, and the crystallization was more refined (average size = 15 nm) with a smoother and denser morphology. Moreover, the Ni/binary-SiC coating presents obviously higher hardness (556 Hv) and higher wear resistance (2.95 mg cm^−2^) than the Ni/SDS-SiC coating. In addition, the Ni/binary-SiC coating exhibited better corrosion resistance. Compared with previous reports, we provided a new insight into the homogeneous dispersion of SiC nanoparticles and the preparation of uniform and high-quality Ni/SiC composite coatings by electroplating.

## Figures and Tables

**Table 1:** Texture coefficient and grain size of composite coatings.

Current Density (A dm^−2^)	Ni/SDS-SiC				Ni/Binary-SiC			
	Texture Coefficient (%)			Grain size (nm)	Texture Coefficient (%)			Grain Size (nm)
	(111)	(200)	(220)		(111)	(200)	(220)	
2.0	14.6	80.2	5.2	32	18.1	10.2	71.7	14
4.0	20.1	72.9	7.0	32	51.8	16.0	32.2	15
6.0	7.9	88.6	3.5	32	50.2	15.2	34.6	16
8.0	8.8	87.5	3.7	31	38.2	15.0	46.8	19

**Table 2:** Electrochemical parameters of the pure nickel, Ni/SDS-SiC and Ni/binary-SiC coatings prepared at the current density of 2.0 A dm^−2^.

Coatings	β_a_ (mV dec^−1^)	β_c_ (mV dec^−1^)	E_corr_ (V)	I_corr_ (μA cm^−2^)	R_p_ (kΩ cm^2^)
Pure nickel	111	622	−0.57	21.99	1.86
Ni/SDS-SiC	228	345	−0.47	8.78	6.79
Ni/binary-SiC	261	221	−0.44	4.74	10.41

β_a_: anodic constant, β_c_: cathodic constant, E_corr_: corrosion potential, I_corr_: corrosion current density, R_p_: polarization resistance.

**Table 3:** EIS parameters obtained by electrical equivalent circuit fitting of the pure nickel, Ni/SDS-SiC and Ni/binary-SiC coatings prepared at the current density of 2.0 A dm^−2^.

Coatings	R_s_ (Ω)	CPE		R_ct_ (kΩ cm^2^)
		Q (Ω^−1^ cm^−2^ s^−n^)	n	
Pure nickel	0.51	1.34 × 10^−4^	0.83	4.29
Ni/SDS-SiC	1.21	3.77× 10^−5^	0.93	11.02
Ni/binary-SiC	0.97	4.08 × 10^−5^	0.93	26.40

R_s_: solution resistance, CPE: non-ideal coating capacity, Q: frequency-independent constant, n: the exponential coefficient, R_ct_: charge-transfer resistance.

## Data Availability

Data is contained within the article.

## Supplementary Materials

The following supporting information can be downloaded at: https://www.mdpi.com/article/10.3390/molecules28083344/s1, Figure S1: Molecular structure of (a) Span 80, (b) Tween 60 and (c) SDS; Figure S2: Fourier-transform infrared spectra of SiC particles before and after modification with Span 80 and Tween 60, respectively; Figure S3: XRD patterns of blank SiC, SD-SiC and binary-SiC; Figure S4: SEM images of (a) blank SiC, SiC dispersed with (b) SDS and (c) binary surfactants; Figure S5: particle size distribution of SiC dispersed with (a) Span 80, (b) Tween 60, (c) Tween 60+ Span 80 and (d) Span 80+Tween 60 (binary surfactant). “+”means consecutive treatments; Figure S6: Grain size distribution diagram obtained by measurement statistics in TEM images. (a) Ni/SDS-SiC coating and (b) Ni/binary-SiC coating prepared at the current density of 2.0 A dm^−2^. Table S1: SiC content of composite coatings; Table S2: Surface roughness of composite coatings.
