# Supplementary material for: Electrodeposition of High-Quality Ni/SiC Composite Coatings by Using Binary Non-Ionic Surfactants
Source: Molecules. 2023 Apr 10;28(8):3344. doi: 10.3390/molecules28083344 (PMC10144436; doi:10.3390/molecules28083344)
Supplement: Supplementary file 1 [file molecules-28-03344-s001.zip › molecules-2317734-supplementary.pdf]

Supplementary Information for

Electrodeposition of high-quality Ni/SiC composite coatings by using binary non-ionic surfactants

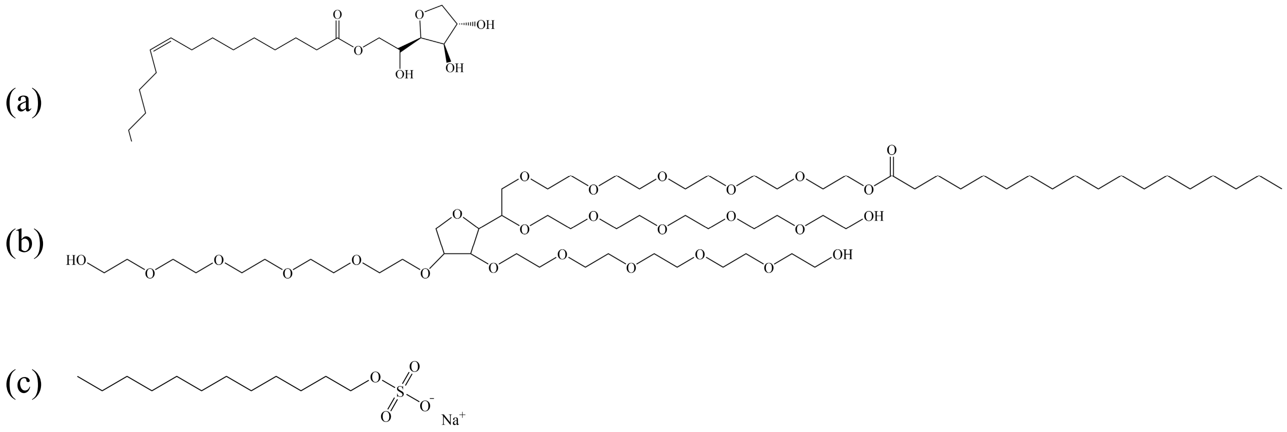

Figure S1 Molecular structure of (**a**) Span 80, (**b**) Tween 60 and (**c**) SDS

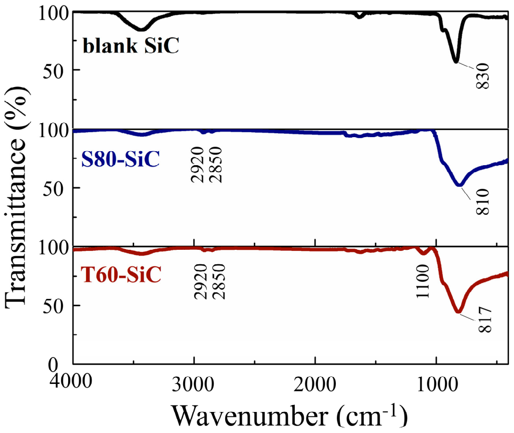

Figure. S2 Fourier-transform infrared spectra of SiC particles before and after modification with Span 80 and Tween 60, respectively

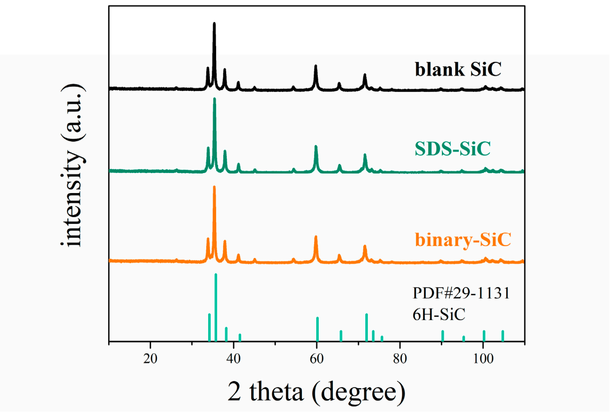

Figure S3 XRD patterns of blank SiC, SD-SiC and binary-SiC

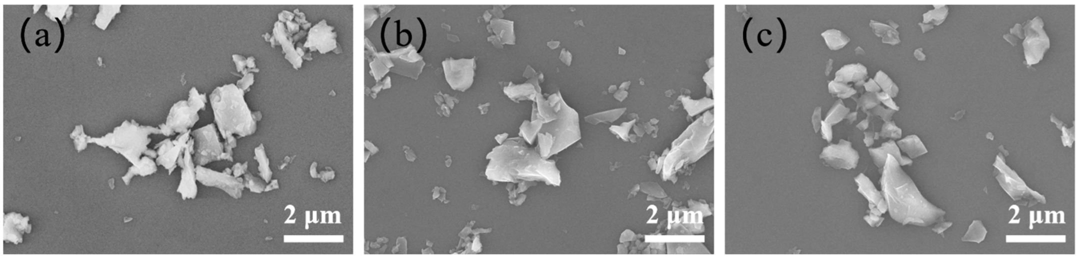

Figure. S4 SEM images of (**a**) blank SiC, SiC dispersed with (**b**) SDS and (**c**) binary surfactants.

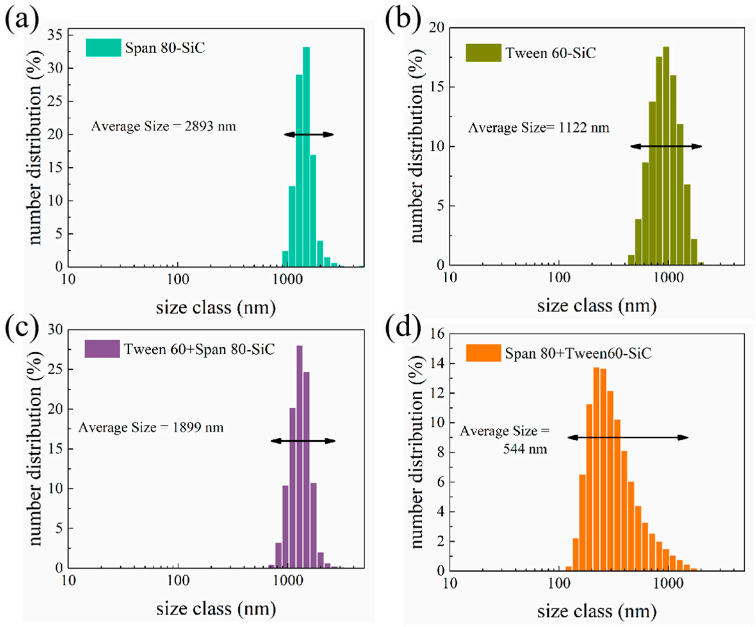

Figure. S5 particle size distribution of SiC dispersed with (**a**) Span 80, (**b**) Tween 60, (**c**) Tween 60+ Span 80 and (**d**) Span 80+Tween 60(binary surfactant). "+" means consecutive treatments.

Table S1 SiC content of composite coatings

Composite coatings	Ni/SDS-SiC				Ni/binary-SiC			
Current density(A/dm^2)	2.0	4.0	6.0	8.0	2.0	4.0	6.0	8.0
SiC content (wt%)	2.7	3.9	1.6	1.1	10.8	9.9	8.4	7.5

Table S2 Surface roughness of composite coatings

Composite coatings	Ni/SDS-SiC				Ni/binary-SiC			
Current density(A/dm^2)	2.0	4.0	6.0	8.0	2.0	4.0	6.0	8.0
Surface roughness (nm)	77	146	111	168	55	62	52	50

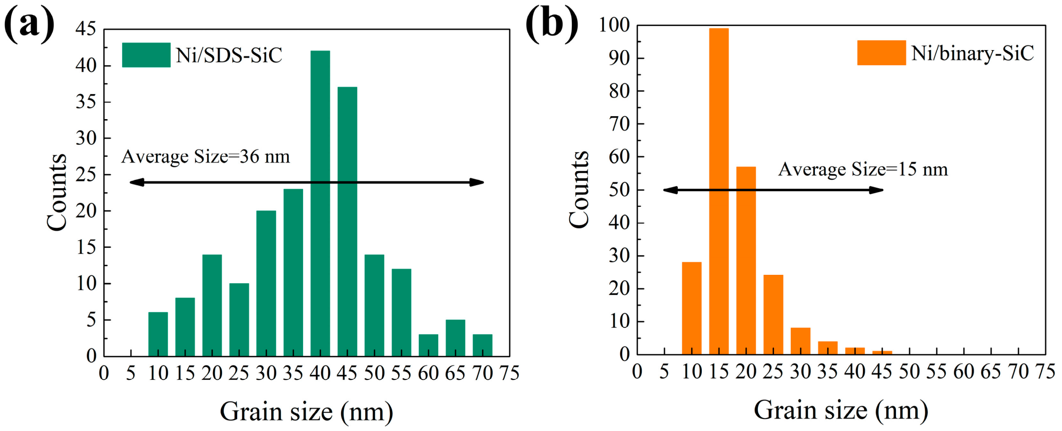

Figure. S6 Grain size distribution diagram obtained by measurement statistics in TEM images. (**a**) Ni/SDS-SiC coating, (**b**) Ni/binary-SiC coating prepared at the current density of 2.0 A dm^{-2}.
